# Sigma metrics in laboratory medicine revisited: We are on the right road with the wrong map

**DOI:** 10.11613/BM.2018.020503

**Published:** 2018-06-15

**Authors:** Wytze P. Oosterhuis, Abdurrahman Coskun

**Affiliations:** 1 Department of Clinical Chemistry and Hematology, Zuyderland Medical Center, Heerlen, The Netherlands; 2 Department of Medical Biochemistry, Acibadem University School of Medicine, Istanbul, Turkey

**Keywords:** biological variation, Six Sigma, Sigma metrics, total error, number of distinct categories

## Abstract

Reliable procedures are needed to quantify the performance of instruments and methods in order to increase the quality in clinical laboratories. The Sigma metrics serves that purpose, and in the present study, the current methods for the calculation of the Sigma metrics are critically evaluated. Although the conventional model based on permissible (or allowable) total error is widely used, it has been shown to be flawed. An alternative method is proposed based on the within-subject biological variation. This model is conceptually similar to the model used in industry to quantify measurement performance, based on the concept of the number of distinct categories and consistent with the Six Sigma methodology. The quality of data produced in clinical laboratories is expected, however, to be higher than the quality of industrial products. It is concluded that this model is consistent with Six Sigma theory, original Sigma metrics equation and with the nature of patients’ samples. Therefore, it can be used easily to calculate the performance of measurement methods and instruments used in clinical laboratories.

## Introduction

The Six Sigma model is accepted as the latest version of total quality management (TQM) and is widely implemented in industry, business and healthcare sector. It is regarded as a powerful management tool and provides specific methods that have the potential to significantly reduce the error rate (*1*). Six Sigma methodology is data-driven and uses a specific problem-solving approach largely based on statistical procedures (*2*). In the Six Sigma model, the Sigma metrics (SM) is the central objective quality measure and the basis for quality control development (*3*, *4*).

According to the Six Sigma theory the performance of a process can be measured easily using the equation given below (Eq. 1) (*2*).


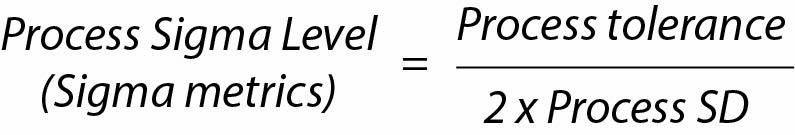


The process tolerance is calculated as the difference (or interval) between the upper and lower tolerance limits (UL and LL, respectively); and SD represents standard deviation.

The Six Sigma methodology was later transferred to laboratory medicine and SM of the measuring process is calculated as given below (Eq. 2),
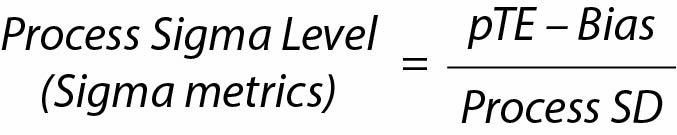
where pTE is the total permissible (or allowable, TEa) error and is calculated as shown in Eq. 3, where k_1_ is the coverage factor (1.65 for 90% probability) and CV_A_ the analytical coefficient of variation (*5*).


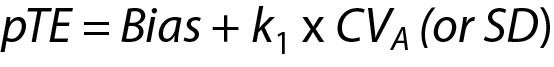


Maximum permissible bias and CV_A_ can be drawn from the data of biological variation as shown below (Eq. 4 and 5),
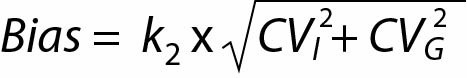


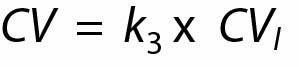
where CV_I_ is the within subject biological variation, CV_G_ the between subject biological variation, and k_2_ and k_3_ are coverage factors. In these equations the coverage factors (k_n_) are variables that depend on the test types and quality requirements.

As shown in Eq. 2, in laboratory medicine the SM is not calculated using the original equation of Six Sigma methodology (Eq. 1), but using a modified equation (Eq. 2). Equation 2 is based on the total error model, where pTE is derived from the estimates of the CV_I_ and CV_G_ (Eq. 3, 4 and 5) using k_n_ coverage factors. The calculation of pTE has, however, been criticized for several reasons, *e.g.* because of the flawed method combining mutually exclusive factors of bias and imprecision (*6*, *7*). Furthermore, the (pTE – Bias) term does not reflect the tolerance limit (TL) concept used in industry.

The calculation of the SM according to the conventional model seems straightforward, but on closer observation there are some serious complications rendering the estimation questionable.

According to the Six Sigma model and Eq. 1, the calculation of the SM requires a TL and not a maximum variation as a limit. With a reliable TL, it is easy to derive analytical performance specifications and also the SM. In the present study, the conventional method to derive a TL from the expression (pTE – Bias) is questioned and an alternative model based on within-subject biological and analytical variations to calculate SM objectively in laboratory medicine is presented. To understand the defects of the model used in laboratory medicine, first of all, we should examine the components of nominator of Eq. 1 and 2.

## Tolerance limit

**Figure 1:**
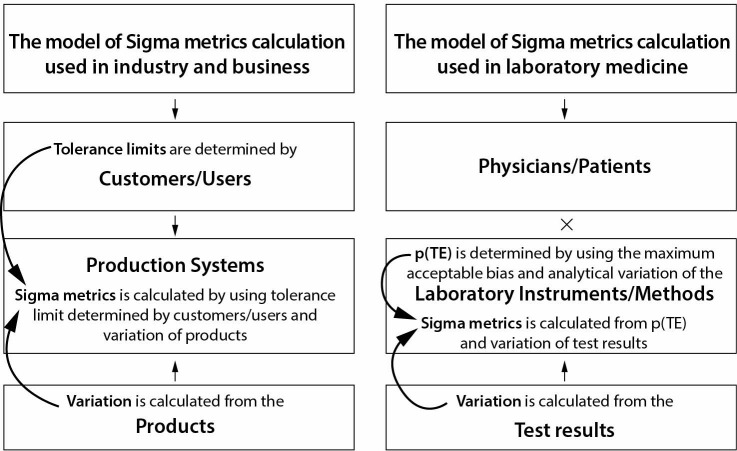
Sigma metrics calculation models used in industry and laboratory medicine. p(TE) – permissible total error.

Tolerance can be defined as the amount of errors or defects that can be tolerated by the customer or user for a given product. The tolerance limit is determined by the customers or users and not by the manufacturer or production system. Therefore, we calculate the performance of a system using the TL determined by the customers/users and deviations obtained from the products. Similarly, in laboratory medicine we should calculate the performance of the instruments/methods using the TL given by the physicians or patients and deviations obtained from the test results (Figure 1).

The equation of the performance used in Six Sigma theory has three components: TL which is determined by customers/users, deviation which is obtained from the products and SM which is the indicator of the quality of the production system. Consequently, the TL should not be determined by the production system or products itself.

In laboratory medicine we use (pTE – Bias) as the TL; however (pTE – Bias) is linked to the instruments and methods. As shown in Eq. 3, 4 and 5, pTE is derived from bias and CV_A_ using coverage factors (k_n_) which is (partly) related to the test types and methods. Both k_2_ and k_3_ depend on the performance of the methods or the number of test results accepted to be defects. When we use quality specifications based on biological variation, k factors become the link between method performance and biological variation. Therefore, in the (pTE – Bias) model, the TL is (in part) a property of the method/instrument and this is a clear contradiction with the Six Sigma concept. Additionally, according to the ‘International vocabulary of metrology’ (VIM 3; 4.26) the term “tolerance” should not be used to designate ‘maximum permissible error’ (*8*).

## Bias as a component of tolerance limit

The second defect of the conventional model is the inclusion of the additional bias in the model used in laboratory medicine.

In the model used in industry, there is no bias in the nominator of the SM equation (Eq. 1). This does not mean that the bias is not present or neglected in the process. Principally, if the bias is present we should correct it. However, in a given time we do not know whether the bias is present or not. Therefore, in the industrial model the presence of 1.5 SD shift is accepted in advance. Nevertheless, for simplicity this shift is not included in the SM equation. Instead, it is included in the conversion table of SM/defects per million opportunities (DPMO). When we convert SM to DPMO we use the table prepared according to short SM-1.5SD. Alternatively, if we measure bias but are unable to correct it, we can calculate SM using z transformation model based on the actual number of results outside the LL and UL (*9*). On the other hand, in the model used in laboratory medicine, in addition to 1.5 SD shift, the measured bias is also included (in fact we need to correct and eliminate it) in the nominator of SM equation, as TEa – Bias. As a result, the SM of conventional model has two biases: the measured bias of the process and 1.5 SD shift. Certainly, in this situation, depending on the measured bias value, the calculated performance of the process will be lower (even extremely lower) than the actual performance (*9*).

## Conventional calculation of Sigma metrics in laboratory medicine

**Figure 2:**
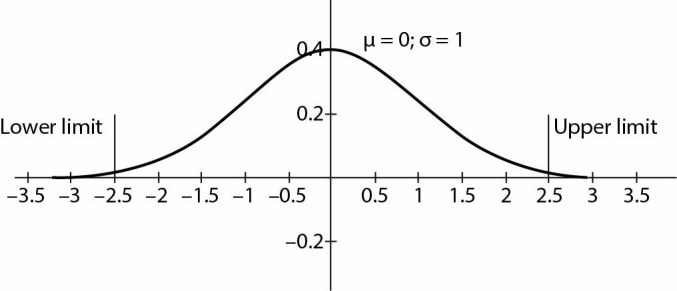
Example of a conventional Six Sigma model with tolerance limits at ± 2.5 standard deviations. μ – mean. σ – variation.

As we mentioned previously, in the Six Sigma model we define upper and lower tolerance limits based on the required product performance. In the example (Figure 2), assume a process with variation (σ) of 1, mean of 0, and TL at ± 2.5 SD:

The observations outside the UL and LL are the defects, counted as DPMO. Outside the 2.5 SD limit will be 0.62% of the observations in case of an assumed Gaussian distribution; for one side DPMO is 6200. According to the Six Sigma reasoning, the quality goal is to improve the production process, bringing down the variance to a level of TLs at 6 SD, not at 2.5 SD as in the example.

In laboratory medicine, analytical performance specifications are in most cases not directly expressed as a TL, but are expressed as maximum CV_A_ (Eq. 6) (*5*).

CV_A_ < 0.5 CV_I_

Generally, the CV_I_ is used to define the performance specification, as this is most applicable when a test is used for monitoring.

Starting with a maximum CV_A_, how to derive the TLs as required in the Six Sigma model for calculation of the SM? By substituting SD for CV_A_, the TL in the conventional model is defined as TEa or pTE.

From the Eq. 1, 2 and 3, the TL used in laboratory medicine can be simplified as given in Eq. 7.


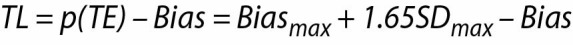


**Figure 3:**
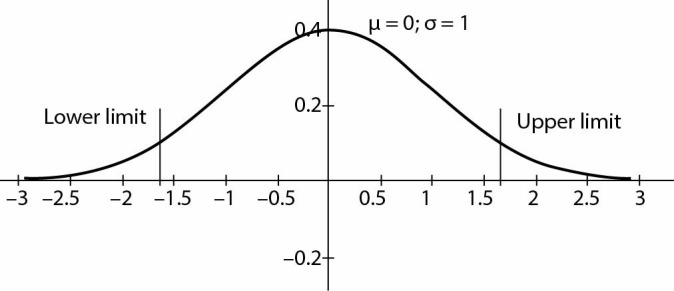
Conventional Six Sigma model in laboratory medicine with tolerance limits at ± 1.65 SD. μ – mean. σ – variation.

The maximum acceptable bias (Bias_max_) and CV_A_ (or SD) can be taken at desirable level (minimum or optimum level depending on test performance) and in this case, Eq. 6 and 7 can be combined and simplified as given below (Eq. 8),


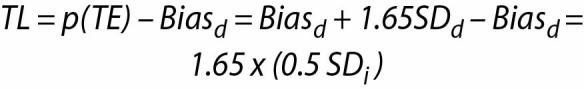


where *d* is the desirable level of analytical specification and SD_I_ the within subject SD. A coverage factor of 1.65 is introduced. With this limit, it is accepted that a maximum of 5% of the results will be outside the LL and UL (Figure 3).

What is the SM of the maximum permissible analytical variation? According to the conventional theory (Eq. 9), with an analytical SD (SD_A_) of 0.5 SD_I_, the SM = 1.65 (*10*).


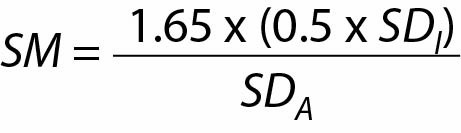


Note the circular reasoning here, as the limit is defined by a percentage of results outside the limit (5%), which defines a sigma score. In other words, in Eq. 9, based on pTE, both nominator and denominator contain the same term 0.5 SD_I_, rendering a result always equal to the coverage factor (in case of SD_A_ = 0.5 SD_I_). Therefore, the SM calculated by Eq. 9, is determined by the coverage factor and cannot predict the performance of measurement methods. As an illustration of this circular reasoning, let us assume another coverage factor, with another percentage outside this limit, *e.g.* pTE = 3.0 (0.5 SD_I_). With a coverage factor of 3.0 it is accepted that only 0.13% of the results will be outside the tolerance limit. What is the SM of this performance? According to the conventional theory,, with a SD_A_ = 0.5 SD_I_, the


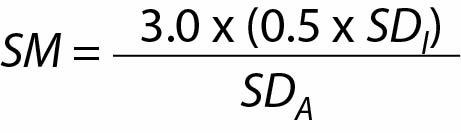


SM = 3.0. Note that we have a higher sigma score with unchanged quality specifications: the maximum SD_A_ still is equal to 0.5 SD_I_. The reason for this equality is, that accepting 5% of results outside the tolerance limit at 1.65 SD is equivalent to accepting 0.13% outside 3.0 SD. This shows that the choice of sigma limit is arbitrary and results in circular reasoning. In the Six Sigma model the process design should result in a minimum number of defects outside the performance limits as possible, preferable < 4 *per* million defining Six Sigma quality. However, here a TL is defined that at the same time accepts as much as 5% “defects”. This seems to contradict the Six Sigma principle.

This leaves the question unanswered how to define the TLs in laboratory medicine based on a maximum SD_A_ of 0.5 SD_I_.

Another approach to this problem might be the internal quality control (IQC) design needed for the different TLs. Obviously, the conventional SM of 1.65 is too low to maintain with quality control, as a SM of at least 3.0 would be needed. If the measurement method performs just within the specification of SD_A_ = 0.5 SD_I_, and the limit of internal quality control was set at 1.65 SD, 5% of the results would be rejected (or 10% two-sided). This would obviously be too impractical for routine use. Therefore, a lower SD_A_ is needed to maintain the pre-defined performance.

Let us assume a SD_A_ that is lower, so the performance could properly be maintained with quality control procedures. In that case, 4.65 SD_A_ should fit within the tolerance limit instead of 1.65.

**Figure 4:**
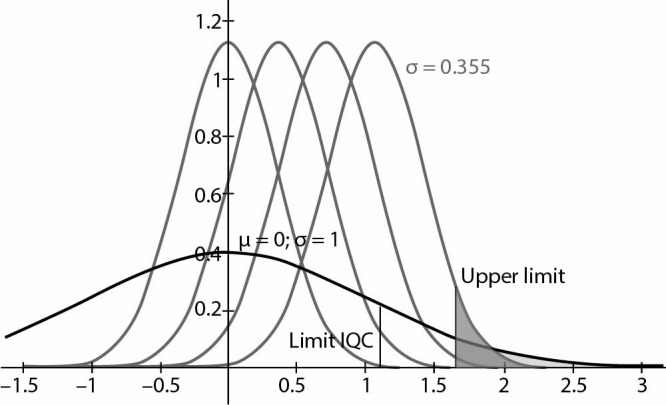
Example of a maximum analytical standard deviation (SD_max_) equal to 0.5 within subject standard deviation (SD_I_); SD as ratio with SD_max_ the actual SD_A_ needed to maintain this performance with quality control procedures is much smaller: SD_A_ = 0.355 SD_max_. μ – mean. σ – variation. IQC – internal quality control.

According to the conventional TEa/Six Sigma model, assuming that the TL (pTE) is at 1.65 (0.5 SD_I_) or 1.65 SD_max_, and we assume 4.65 SD_A_ within this limit, we should have an analytical SD_A_ of 0.35 SD_max_ to properly maintain the required quality. With an IQC limit at 3.0 SD_A_ (equal to 1.1 SD_max_) we still have the required 1.65 SD_A_ safely margin. In case of a shift (bias) of 1.65 SD_A_ we have an error detection of 9% (singular measurement) and only an expected 0.13% of the results outside the TL. In case of a larger shift of 3.0 SD_A_ we have an error detection of 50%, with 5% of results outside the TL. This 5% outside the 1.65 SD_A_ limit is equal to the 5% outside this limit of the 0.5 SD_I_ curve (Figure 4). The question is what is the SM of this system? According to the conventional model,, with SD_a_ = 0.355 (0.5 SD_I_)
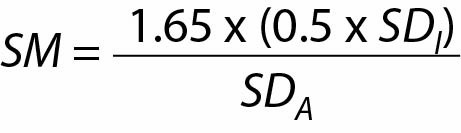

the SM = 4.65.

**Figure 5:**
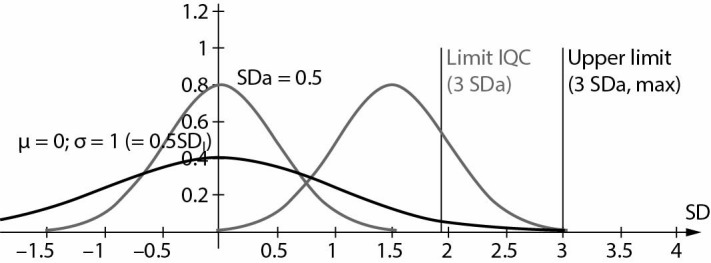
Maximum analytical standard deviation (SD_max_) is equal to 0.5 within subject standard deviation (SD_I_), with IQC limit at 1.94 SD_max_ and tolerance limit at 3.0 SD_max_. X-axis: SD as ratio to SD_max_. μ – mean. σ – variation.

Alternatively, we could choose an IQC model with a TL at 3.0 SD corresponding to 0.13% of results outside the limit, instead of 1.65 SD with 5% outside the limit (Figure 5). Assuming that the TL is at 3.0 SD_max_ and we again assume 4.65 SD_A_ within this limit, we should have an analytical SD_A_ of 0.65 SD_max_ to properly maintain the required quality. With an IQC limit at 3.0 SD_A_ (1.94 SD_max_) there still is a 1.65 SD_A_ safely margin. In case of a shift (bias) of 1.65 SD_A_, the error detection is again 9% and expected 0.13% of the results outside the TL. In case of a larger shift of 3.0 SD_A_ (1.94 SD_max_) we have an error detection of 50%, with 5% of results outside the limit.

The SM of this system is,
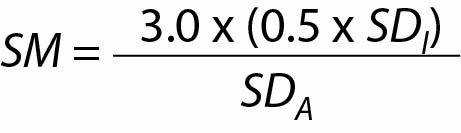
and with SD_A_ = 0.645 (0.5 SD_I_) = 0.323 SD_I_, the SM = 4.65

Both the TL and SD_A_ were increased in proportion, resulting in an unchanged SM of 4.65. The problem how to define the TL has now been shifted to the problem how to maintain the required SD_max_ by IQC. Note that a coverage factor of 3.0 is generally accepted when maintaining a certain analytical variation, so the first coverage factor of 1.65 could be regarded as too strict (*11*).

## Measurement theory in industry

The Six Sigma concept originates from industry, first introduced by Motorola in 1986. Methods for quality control of manufacturing processes have many concepts in common with those in laboratory medicine. In industry, products should fulfil the pre-set quality specifications and measurement procedures should be of sufficient accuracy and precision to distinguish good from bad products. The methods of measurement procedures of the Automotive Industry Action Group (AIAG) are often referred to when the specifications of measurement system analysis in industry are described (*12*). In automobile industry, the quality requirements are high: a component with a quality below limits could lead to substantial financial loss. Although the Six Sigma model is broadly applied in production processes, it should be noted that in contrast to laboratory medicine, the quality of measurement according to the AIAG is not expressed as SM but as ‘Number of Distinct Categories’ (NDC).

The criteria as to whether a measurement system’s variability is satisfactory depends upon the manufacturing production process variability in relation to the measurement system variability. The NDC refers to the number of distinct (non-overlapping) categories that can be distinguished by a measuring system in relation to the variability of the product to be measured. Following this procedure, the ratio of the process (or part) variability to the variation of the measurement system (named repeatability and reproducibility) is calculated (Eq. 10).


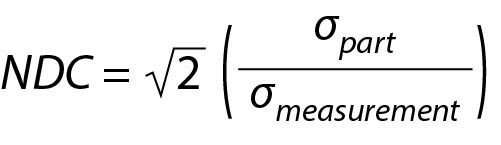


See Table 1 for a common interpretation of these ratios. Central here is the concept of NDC (AIAG).

**Table 1:** Decision-making based on the number of distinct categories used in automotive industry

**NDC**	**Decision**	**Comments**
**> 14**	Generally considered to be an acceptable measurement system	Recommended, especially useful when trying to sort or classify parts or when tightened process control is required.
**4 - 14**	May be acceptable for some applications	Decision should be based upon, for example, importance of application measurement, cost of measurement device, cost of rework or repair. Should be approved by customer.
**< 4**	Considered to be unacceptable	Every effort should be made to improve the measurement system. This condition may be addressed by the use of an appropriate measurement strategy; for example, using the average results of several readings of the same part characteristic in order to reduce final measurement variation.
NDC - the number of distinct categories that can be distinguished by a measuring system in relation to the variability of the product to be measured.		

## Alternative Sigma metrics calculation in laboratory medicine

**Figure 6:**
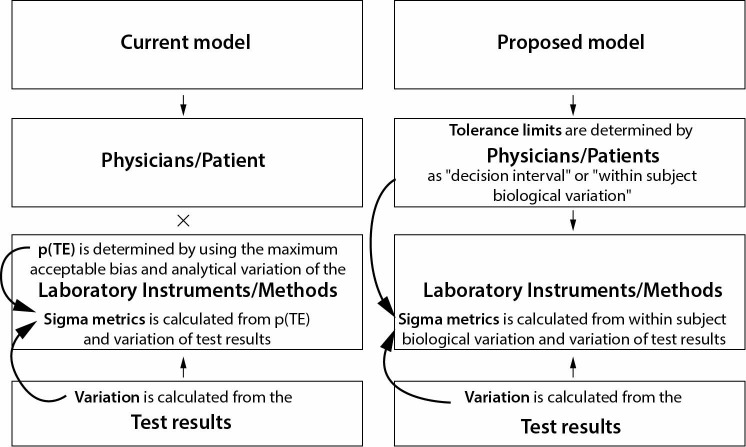
The current and proposed model to calculate sigma metric in laboratory medicine. p(TE) – permissible total error.

We propose a model to calculate the SM that is consistent with both Six Sigma model and the quality principles in laboratory medicine. As shown above the main discrepancies between the model used in industry and laboratory medicine is the TL. We need a reliable TL which is consistent with both Six Sigma theory and laboratory facts. The new model is illustrated in Figure 6 and Table 2.

**Table 2:** Calculation of Sigma metrics according to different models compared to the number of distinct categories

**Test**	CV_A_* (%)	**CV_I_ (%)**	**CV_G_ (%)**	**pTE^†^ (%)**	**SM_pTE_**	**SM_BV_**	**NDC**
**Creatinine**	2.6	5.9	14.7	8.9	3.5	2.3	3.3
**Sodium**	1.1	0.6	0.7	0.7	0.7	0.54	0.8
**Potassium**	1.4	4.6	5.6	5.6	4.1	3.38	4.9
**Glucose**	0.7	5.6	7.5	7.0	9.9	8.0	11.3
**Iron**	1.8	26.5	23.2	30.7	17.3	15.0	21.2
**Albumin**	2.6	3.2	4.75	4.07	1.6	1.2	1.7
**TSH**	1.2	19.3	24.6	38.2	30.6	15.4	21.8
CV_A_ - analytical variation. CV_I_ – within-subject biological variation. CV_G_ – between-subject biological variation. SM_pTE_ – Sigma metrics (SM) derived from permissible total error (pTE). SM_BV_ - Sigma metrics derived from (within-subject) biological variation (CV_I_). *Values obtained from local laboratory as example. **^†^**Conventional TE model SM_pTE_: [0.25 (CV_I_^2^ + CV_G_^2^)^0.5^ + 1.65 (0.5 CV_I_)] / CV_A_ (*14*). Alternative model, SM_BV_ = CV_I_ / CV_A_. NDC – number of distinct categories; 1.41 (CV_I_ / CV_A_); note that the limit is 4, while a minimum SM is generally considered to be 3.							

In the new model, we thought that the TL of analytes being measured in clinical laboratories should be consistent with the hierarchy of models of Milan consensus as given below:

Model 1. The effect of analytical performance on clinical outcomes;
Model 2. The biological variation of the measurand;
Model 3. State-of-the-art analytical performance of the measurement (*13*).

Decision interval and/or within subject biological variation can be accepted as the TL. This approach is consistent with the Six Sigma theory. Although Model 1 is the preferred model, it has limited application since this needs a well-defined decision limit. On the other hand, the biological variation of the measurand is more practical and can be applied in most cases. Within subject biological variation is the inherent variation of the measurand and directly related to patients’ metabolism and not to the methods and/or instruments of the laboratory. Therefore, it can be used as the TL of the measurand.

In the proposed model, the SM of the process is estimated as a ratio of CV_I_ of the measurands to the measurement (analytical) variation (CV_A_). Principally it is similar to SM calculation in industry and the model of NDC. In this model, we do not use a coverage factor such as 1.65.

The model can be formulated as given below (Eq. 11).


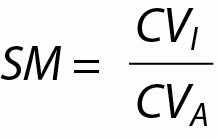


To maintain the quality, we need SM = 4.65; therefore
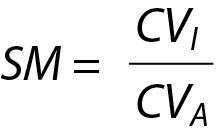
and CV_A_ = 0.22 CV_I_.

## Discussion

We have shown that it is complicated to derive a reliable SM based on a maximum analytical variation according to the common definition CV_A_ < 0.5 CV_I_. In our opinion, the conventional definition of the SM based on pTE is flawed due to circular reasoning, and is arbitrary to a high degree.

The main problem of SM calculation in laboratory medicine is the lack of a reliable method for the estimation of TLs. These are essential not only for SM but also for quality specifications and development of IQC methods. Therefore, various attempts have been made to find a solution for a consistent procedure specific to laboratory medicine such as Milan and Stockholm consensus (*13*, *15*).

We proposed that the CV_I_ of measurands could be accepted as the TL of the measurands due to the following reasons:

1. Biological variation originates from patients and reflects the natural variation of measurands;
2. Biological variation data are not method and/or instrument dependent;
3. It is consistent with Six Sigma theory and SM equation (Eq. 1 to 11);
4. It has no coverage factor such as pTE and not influenced by the bias as the current model used in laboratory medicine;
5. It is test specific and does not vary among laboratories. Therefore, it could be preferred as the reference for harmonization of the measurement of instruments/methods performance.

The presented model, in a way is also similar to the NDC concept that is being used in automotive industry. The advantage of NDC model is that no TL is needed and this model is not prone to errors such as the conventional model based on pTE. Additionally, in this model we do not use various coverage factors such as 1.65 or 3 to exclude a given percentage of data, which makes TL defective.

If we define a maximum permissible analytical variation based on the CV_I_, it should be noted that there is a corresponding analytical variation that is more strict and will serve as the actual maximum analytical variation applied in IQC and that will include a safety margin in order to maintain the maximum permissible variation with a predefined probability. According to the proposed model, the ratio SD_A_ / SD_I_ (or CV_A_ / CV_I_) would be 0.22 for the required 4.65 Sigma metric.

In classical Six Sigma theory, despite the pre-acceptance of 1.5 SD shift (as in “long term” SM), the defect rate is less than 4 DPMO. It should be noted that Six Sigma originated from industry and not from the healthcare sector. Currently, the expected quality level required in automotive or aviation sectors are higher than even 6 sigma. However, we cannot assume that the desired quality level in laboratory medicine is lower than in the automotive or aviation sectors. The 3 sigma quality level, that is the accepted minimum quality for laboratory medicine, is far away from the quality of industrial products. To decrease laboratory errors to a negligible level we should investigate new and sound models to evaluate the performance of measurement methods objectively.

The proposed model does not take bias into account. This is not a defect of the model. Remember that in the Six Sigma theory (Eq. 1) we also do not include bias directly to the equation of SM, because we use the SM/DPMO conversion table prepared according to the short SM - 1.5 SD. Additionally, it has been stated that many forms of short-time bias are being included in the intermediate analytical imprecision and can be regarded as un-correctable (*7*). It can be argued that other forms of bias that are correctable (*e.g.* due to bias in standards higher in the traceability chain) should not be expressed in the SM as quality measure on the level of the individual laboratory.

Although consensus has been reached to accept the biological variation as basis for calculation of performance specifications, two major issues of biological variation must be addressed. Firstly, the published biological variation data vary significantly and may not always be reliable; and secondly, data of biological variation are not available for all measurands. Recently the European Federation of Clinical Chemistry and Laboratory Medicine (EFLM) Biological Variation Working Group initiated the European Biological Variation Study (EuBIVAS) and collected samples from six European countries following a stringent protocol (*16*). So far, biological variation estimates for several measurands have been determined and others are in preparation (*17*-*19*). An update of the biological variation database has been planned and will be made available.

In conclusion, this study shows that the conventional method for calculation of the SM is flawed. An alternative method for calculation of the SM, which is consistent with both Six Sigma theory, and concept of performance specifications based on biological variation is presented. This new method is simple and can be used by laboratory staff easily.
